# Miscellany

**Published:** 1901-06

**Authors:** 


### Miscellany.

WEIGHTING A FULL LOWER DENTURE.—When I once thought it necessary to increase the weight of a lower set of vulcanite, I simply packed inside the rubber small strips of lead or tin. I have long since ceased to realize any special benefit from extra weight. For many years I advocated the use of cast-metal plates. A few years since, I was obliged to resort to a full denture. For my second plate, as the jaw is very flat and narrow, I used a cast-metal plate. To my surprise I found it would not answer at all. There was no ridge to prevent its sliding forward or sidewise. If I leaned forward to speak to a student, the plate slid forward. Upon lying down in bed, it would slide sidewise.—L. P. HASKELL, *Dental Brief*.

---

HOW TO LIGHTEN FULL UPPER CASES WITH SIDE-BLOCKS.—In the first place, do not use the blocks. Plain teeth can be articulated far better. But why wish to lighten them? Weight cuts no figure in a properly adjusted upper denture. I do not consider it a factor in any case whatever. As evidence, I have been putting in continuous gum dentures for fifty years, and find weight no objection, and have constantly replaced rubber plates with them, and no complaint comes of weight.—L. P. HASKELL, *Dental Brief.*

---

POSSIBLY A VALUABLE DRUG.—An Indian medical journal publishes a description of a curious plant which grows in Arabia and parts of the western frontier of Hindustan. It is popularly known as "the laughing plant," on account of the effect produced by eating the seeds. The plant is of moderate size, with bright yellow flowers and soft, velvety seed-pods, each of which contains two or three seeds resembling small black beans. The natives of the district where the plant grows dry these seeds and reduce them to powder. A small dose of this powder has similar effects to those arising from the inhalations of laughing-gas. It causes the soberest person to dance, shout, and laugh with the boisterous excitement of a madman, and to rush about and cut the most ridiculous capers for about an hour. At the expiration of that time exhaustion sets in, and the excited person falls asleep, to wake after several hours with no recollection whatever of his antics.—*Public Health Journal.*

---

A NEW METHOD OF MAKING DUMMIES.—After the abutments to which the dummies of a bridge are to be attached are made, porcelain teeth are ground into place to articulate with the opposing teeth. The porcelain teeth thus articulated are waxed together, and an impression of them is taken in mouldine, and a die is poured, upon which No. 28 gold plate is swaged directly into lead, using no counter-die. A mould in gold thus formed is flowed solidly full of solder or is backed up with gold and soldered. This is then filed up to fit between the two crowns already formed for the bridge and the whole soldered together, and thus a solid gold bridge is obtained.— DR. J. K. SMITH, *Dental Register.*

---

THE URIC ACID DIATHESIS.—James Tyson holds that the uric acid diathesis is remediable in two ways,—first, by limiting the ingestion of nitrogenous food, whence uric acid and its congeners are derived; secondly, by increasing the quantity of the water constituent of urine and by alkalizing the blood and urine with the view of favoring solution and elimination of uric acid. There is no contraindication to the use of carbohydrates and hydrocarbons, provided they do not produce acid fermentation. Nor is there any objection to the use of fruits which are not acid. Exercise, bathing, and massage should also constitute part of the treatment. The alkaline bases should be administered, principally the salicylates, the citrates, and bicarbonates.—*Medical Record.*

---

ALCOHOL AS AN ANTISEPTIC.—The *Hospital* has this to say in regard to alcohol as a disinfectant, particularly for the hands. The article gives the results of experiments of Salzwedel and Elsner, made in Koch's laboratory. "They maintain that these researches show that alcohol is of use in preparing the hands for operations, not merely because of its hardening effect on the epidermis, but also as an active antiseptic; in this respect they assign it a potency intermediate between carbolic acid and corrosive sublimate."—*Medical Record.*

---

TO REPAIR A FRACTURED PORCELAIN FACING ON A FIXED CROWN OR BRIDGE.—I forget to whom I am indebted for the method, but I use little platinum tubes of the diameter of a pin. The pins are cut off close to the porcelain and the tubes are soldered on with pure gold. Two holes, corresponding to these tubes, are drilled through the backing and the tooth is tried in. The length of the tubes is marked off, the tooth removed, and the tubes cut off. The tooth is then replaced with cement and fixed in position with an Ainsworth punch, by pressing the tubes so that they form a rivet. The inside of the tubes is packed with amalgam or gold. The efficacy of the method is remarkable. I have used this method of repair since it was given three years ago, and it is very convenient.—DR. D. HEADRIDGE, *Journal of the British Dental Association.*

[If a bevel is given to the inner ends of the holes, the ends of the tubes can be made to form rivets, and, besides, it will facilitate their being filled with either gold or amalgam.—J. A. McC.]

METHOD OF SAVING A BADLY DECAYED AND FRACTURED ROOT.—At the eighth annual clinic of the Alumni Association of the Chicago College of Dental Surgery, Dr. Keefe, of Chicago, gave a clinic illustrating the method of saving a badly decayed and fractured root by extracting, preparing, and banding outside the mouth and then replanting. The tooth treated was an upper right lateral, which was badly decayed, and fractured upon the labial side to the process. After extracting, the canal was cleaned and filled with gutta-percha and a silver band fitted to the cervical end, as would be done for a crown. The band was made wide enough to reach the margin of the gum. The root was then replanted and will be crowned when the parts have assumed a healthy condition.

Dr. Keefe exhibted several X-ray illustrations of other teeth replanted during the past eight years for similar reasons.—*The Bur.*

---

KOWARSKA'S PASTE.—Kowarska's paste is composed of celluloid, one hundred and fifty-five grains, and acetone, five hundred grains. It is used to retain loose teeth in position and to cover the sensitive necks of teeth. The method of Dr. Hinkins, of Chicago, in the use of Kowarska's paste for holding loose teeth in position is described in the *Dental Review* as follows:

"This procedure is to ligate the teeth together, not in the figure-of-eight style, but by tying the silk about the tooth and making several knots with silk, enough to fill nearly the interdental space; then to encircle the next tooth and tie knots again in the interdental space, and so on, until all the loose teeth are secured. Then with this cement, which is made of celluloid C. P. and acetone C. P. dissolved together to make a thick creamy mass, the parts are held firmly together. Apply the cement about twice as thick as desired, because it shrinks."

No doubt other uses will be found for this paste, but it must be kept in mind that it will become foul like all preparations of celluloid, much more so than when prepared in the usual manner for plates.

---

ALUMINUM VERSUS GOLD FOR CLASPS.—The teeth embraced by gold clasps are especially prone to decay on the surface covered by the clasp. For nine years I have frequently used aluminum for clasps, and not one has caused any trouble. Not once have I seen any bad results from the use of this metal.—H. R. NEEPER, *Dental Digest.*

[The above is taken from the *Pacific Dental Gazette.* In regard to the use of aluminum for clasps, it lacks the essential quality of clasp metal,—namely, elasticity. Besides, the loss of tooth-structure under a clasp is caused by chemical solution, not mechanical abrasion. The food deposits beneath and about the clasp undergo lactic fermentation, and the lactic acid there formed dissolves the tooth-structure. The preventive measure is absolute cleanliness, hence should be as likely to occur under an aluminum clasp as under a gold one.—J. A. McC.]

---

A FLUID FLUX THAT DOES NOT PIT.—This consists of a saturated solution of equal parts of boracic acid and borax. It is prepared in the following manner:

Mix equal parts of boracic acid and powdered borax and place them in sufficient water to get a saturated solution. This may be determined by a slight residue on the bottom of the receptacle.—DR. DODEL, *Ohio Dental Journal.*

---

TO HOLD INLAYS WHILE CUTTING THE GROOVE.—In cutting the groove around an inlay before cementing it in the cavity, much difficulty is experienced in holding the very small inlays. This can be overcome by sticking the inlay to the end of a piece of orange-wood with shellac, which holds it firmly and permits the operator to cut the groove readily.—C. F. ALLEN, *Dental Review.*

---

USE OF RUBBER DAM IN INLAY WORK.—The greatest help we have had recently in inlay work is the method, introduced by Dr. Reeves, of using rubber dam or any similar substance for holding the matrix to the cavity during the final burnishing.—C. N. THOMPSON, *Dental Review.*

